# Python-Based Open-Source Electro-Mechanical Co-Optimization System for MEMS Inertial Sensors

**DOI:** 10.3390/mi13010001

**Published:** 2021-12-21

**Authors:** Rui Amendoeira Esteves, Chen Wang, Michael Kraft

**Affiliations:** MNS, Department of Electrical Engineering (ESAT), University of Leuven, 3001 Leuven, Belgium; resteves@esat.kuleuven.be (R.A.E.); michael.kraft@kuleuven.be (M.K.)

**Keywords:** microelectromechanical systems (MEMS), inertial sensors, Python, finite element method, genetic algorithm, optimization, accelerometer, gyroscope

## Abstract

The surge in fabrication techniques for micro- and nanodevices gave room to rapid growth in these technologies and a never-ending range of possible applications emerged. These new products significantly improve human life, however, the evolution in the design, simulation and optimization process of said products did not observe a similarly rapid growth. It became thus clear that the performance of micro- and nanodevices would benefit from significant improvements in this area. This work presents a novel methodology for electro-mechanical co-optimization of micro-electromechanical systems (MEMS) inertial sensors. The developed software tool comprises geometry design, finite element method (FEM) analysis, damping calculation, electronic domain simulation, and a genetic algorithm (GA) optimization process. It allows for a facilitated system-level MEMS design flow, in which electrical and mechanical domains communicate with each other to achieve an optimized system performance. To demonstrate the efficacy of the methodology, an open-loop capacitive MEMS accelerometer and an open-loop Coriolis vibratory MEMS gyroscope were simulated and optimized—these devices saw a sensitivity improvement of 193.77% and 420.9%, respectively, in comparison to their original state.

## 1. Introduction

The design, simulation, and optimization process of a MEMS device include a sequence of designing the initial geometry, mechanical parameter simulation and optimization, designing of the electrical interface, and simulation of the complete system [1].

To design, simulate, and optimize MEMS inertial sensors, engineers typically separate the process in two very distinct—yet symbiotic—domains: mechanical and electrical domains. This workflow is often strictly divided and a great deal of simplification is applied to one of the domains in order to allocate computational resources to achieve a complete simulation and optimization of the other [2].

MEMS mechanical structures are designed with a computer-assisted design (CAD) software and commonly comprise thousands of degrees of freedom (DoF) which lead to a heavy computational cost when simulating mechanical behavior. To bypass this obstacle, engineers have used reduced-order modelling methods to build system-level models [3,4,5]—bringing the thousands of DoFs down to a few, frequently used when designing closed-loop control systems [6]. Younis et al. [7] developed a reduced-order model to study the behavior of electrostatically actuated microbeams-based MEMS, using a macro model to reduce the computational time. The aforementioned method can be helpful when designing the sensor electrical interface, but it fails to take into consideration the full complex mechanical structure, and consequently the interaction between electrical and mechanical domains.

Moreover, the typical optimization methodology combines multiphysics software and a programming language interpreter program. Wang et al. [8,9] presented a MEMS mechanical optimization method that allows for the generation of freeform geometries—combining COMSOL [10] finite element analysis and modelling with a GA implemented in MATLAB [11], demonstrating its effectiveness with the optimization of a MEMS accelerometer comprising a mechanical displacement amplifier.

Nevertheless, this type of approach has several limitations. Firstly, it does not fully capture the interaction between mechanical and electrical domains. In addition, there is a very restricted set of tools to choose from, combined with the need for compatibility between different commercial software. That severely limits the potential for customization and adaptation to specific designs. Moreover, the high licensing costs for both COMSOL Multiphysics software and MATLAB turn this method into unreachable tools for many researchers. The open-source nature of all parts composing this software transforms it into a free, fully customizable solution.

In this work, a Python-based [12] open-source co-optimization tool for MEMS inertial sensors was demonstrated. The developed software combined geometry design, FEM analysis, viscous damping calculation, electronic domain simulation, and a GA that takes into account all aforementioned blocks. In this way, a robust optimization tool comprising mechanical, electrical, and damping parameters, was achieved and used to improve the overall system performance.

## 2. Tools and Methods

### 2.1. Finite Element Method (FEM)

The finite element method was implemented in the proposed co-optimization system to simulate complex mechanical geometries.

The FEM approaches any simulation problem by subdividing a continuous entity into finite smaller parts, solving each one individually, and reassembling them. Many physical processes, and especially most solid mechanics ones, can be described by partial differential equations (PDEs). For a computer to solve PDEs, it applies the FEM to divide a complex system into smaller subparts—finite elements. This division process is called space discretization, and the generation of a mesh. This is a way of transcribing a 2D or 3D object into a series of mathematical points that can be analyzed. For static solid mechanics studies, the main category of PDEs are elliptic, which can be solved using a variational FEM method [13]. A variational method has its basis on the principle of energy minimization: when a boundary condition is applied, the configuration where the total energy is minimum is the one that prevails. This process starts with the multiplication of PDEs by a test function, then integrate the resulting equation over the domain, and finally perform integration by parts with second-order derivatives [13].

### 2.2. Python Language and Libraries

The Python programming language was used to develop the co-optimization system in this study. It is a high-level language and well suited for scientific and engineering environments. Its highly modular nature and clean syntax provide a simple and direct code writing suitable in many scientific applications [14]. The main advantage of Python language lies in the countless number of library modules provided by either official Python or the global developer’s community, which offers numerous potential combinations and applications.

### 2.3. Python Simulation and Optimization Software

A complete MEMS co-simulation and co-optimization program comprises different essential blocks. This type of software needs a geometry designer and processor with meshing abilities, a FEM simulation block powerful enough to process different mesh sizes with varying degrees of complexity, a dedicated electrical domain script capable of interpreting the mechanical results of each MEMS design, and a GA that takes into consideration both mechanical and electrical performance parameters.

**Figure 1:**
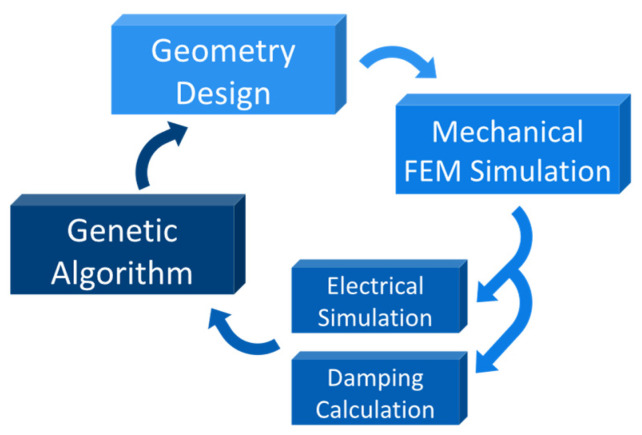
General block diagram for the developed software.

A software covering all the aforementioned blocks was developed with a general structure depicted in Figure 1. The software tool also comprised a viscous damping calculation. In this way, the optimization considers an additional important performance parameter.

#### 2.3.1. MEMS Geometry Design in Python

A competent geometry design and meshing tool require several fundamental characteristics: the ability to create different shapes and perform Boolean operations on them, the capacity to generate a customizable mesh to be assigned to the created geometry, and the possibility to import and export files. Two Python libraries were chosen—*pygmsh* [15,16] and *meshio* [17]. *Pygmsh* is used for geometry building and mesh generation, the *meshio* library is applied to generate a file that can be read by the other software blocks.

#### 2.3.2. FEM Simulation for Displacement and Modal Analysis

The FEM is employed in this tool to solve PDEs problems involving linear elasticity equations: the first is to calculate the displacement resulting from an applied force, and the second is to perform a modal analysis—obtaining the MEMS eigenfrequency modes.

When a force is applied to a body Ω, the equations describing the related deformations of the geometries with isotropic elastic conditions are described in [18].

To study MEMS inertial sensors, the knowledge of natural frequencies and the corresponding mode shapes are essential. Modal analysis solves an eigensystem—a group of all eigenvectors belonging to a linear transformation matched with the corresponding eigenvalue. In an eigensystem, the eigenvector represents the mode shape and the eigenvalue represents the frequency.

#### 2.3.3. Electronic Domain Simulation

The electronic domain simulation block is the third. In the readout circuit, the number of comb fingers and overlapping area varies with the geometry, and the actuation scheme changes for each design. Two MEMS inertial sensors were simulated, i.e., a capacitive accelerometer and a linear vibratory gyroscope. The MEMS accelerometer has a proof-mass between two electrodes, which results in differential sensing defined by Equation (2). The MEMS vibratory gyroscope has a proof-mass between two sets of comb-fingers which moves along the *y*-axis, modifying the gap between the fixed electrodes and the moving ones as described by Equation (4). In this group of equations, *N* stands for the number of comb fingers, *t* is the thickness, *L* represents the comb fingers length, and *d* signifies the gap between the fingers.

(1) $$C_{top}=\frac{\epsilon_{0}\epsilon_{r}a}{d-disp},\;\;C_{bot}=\frac{\epsilon_{0}\epsilon_{r}a}{d+disp},$$

(2) $$\Delta C=C_{top}-C_{bottom},$$

(3) $$C_{top}=\frac{\epsilon_{0}\epsilon_{r}tL}{d-disp},\;\;C_{bot}=\frac{\epsilon_{0}\epsilon_{r}tL}{d+disp},$$

(4) $$\Delta C=C_{top}-C_{bottom}\;\approx 2N\frac{\epsilon_{0}tL}{d^{2}}disp$$

To drive the gyroscope’s proof-mass into resonance, electrostatic actuation is applied. Electrostatic actuators rely on the force between two electrodes when a voltage is applied between them [19]. Parallel-plate actuation electrodes are commonly built to apply a force in a specific direction—aligned with the desired motion direction and DoF of the target mass.

In the designed MEMS gyroscope, a balanced actuation mechanism was applied. The actuating comb-fingers generate the desired actuation force by sliding parallel to each other, as described in Equation (5)—a potential $V_{1}=V_{DC}+V_{AC}$ is applied to one set of electrodes and another potential $V_{2}=V_{DC}-V_{AC}$ to the opposing set.

(5) $$F_{balanced}=2\frac{\epsilon_{0}LtN}{d^{2}}\;V_{DC}V_{AC}$$

The calculation of the sensing and actuating mechanism is included in the software to study the influence of electrical parameters on the device performance through the genetic algorithm and thus achieving electro-mechanical co-optimization.

**Figure 2:**
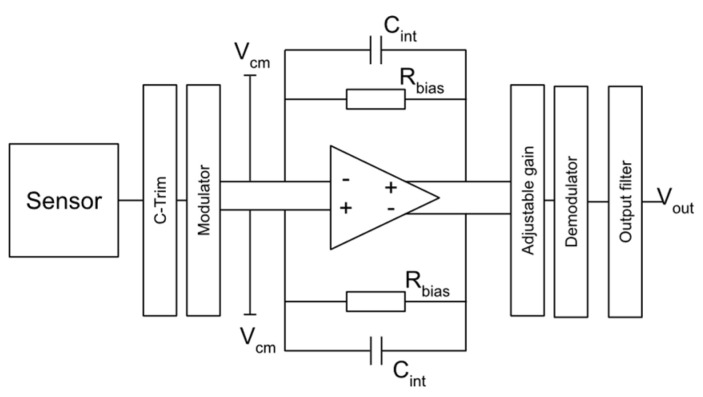
Block diagram of capacitance-to-voltage converter circuit implemented with both MEMS devices.

A capacitance-to-voltage converter was implemented in the code, to calculate a voltage output from the acceleration induced capacitance change. The converter is based on the converting block designed by Utz et al. [20], as shown in Figure 2 with a governing equation stated in Equation (6), in which *V_DD_* is considered 5 V and *V_CM_* is considered 2.5 V.

(6) $$V_{C2V}=\frac{2\Delta C_{S}\left(V_{DD}-V_{CM}\right)}{C_{int}}$$

#### 2.3.4. Damping Calculation

In this work, the damping was modeled as viscous damping, which is the primary contributor to overall damping of the system. This type of damping mechanism occurs when the gas surrounding the vibratory structures introduces viscous effects caused by the internal friction of the gas trapped in the middle of vibratory structures such as comb fingers. Here, two viscous film damping effects, i.e., squeeze film damping and slide film damping, were modelled to calculate the quality factor of the drive and sense modes for both the accelerometer and gyroscope. The simulated MEMS inertial sensors were considered to be surrounded by air (ε_r_ = 1) at the atmospheric pressure of 1013.25 mbar.

To model the two viscous film damping effects, the following equations were implemented [21,22]:

(7) $$K_{n}=\frac{\lambda_{f}}{d}$$

(8) $$\mu_{eff_{squeeze}}=\frac{\mu }{1+9.638\cdot K_{n}^{1.159}}$$

(9) $$\mu_{eff_{slide}}=\frac{\mu }{1+2\cdot K_{n}+0.2\cdot K_{n}^{0.788}\cdot e^{-K_{n}/10}},$$

(10) $$c=N_{E}\cdot \mu_{eff}\cdot l_{E}\cdot {\left(\frac{h_{E}}{d}\right)}^{3}$$

(11) $$Q=\frac{M\cdot \omega }{c}$$

In Equation (7), *K_n_* represents the Knudsen number which is a measure of gas rarefaction effect. *λ_f_* is the ratio of the mean free path and *d* refers to the gap containing the gas. This equation makes the ambient pressure of the sensor’s working environment. Equation (8) denotes the effective viscosity of squeeze film damping in which *µ* stands for the mean viscosity. Equation (9) describes the effective viscosity of slide film damping. Equation (10) refers to the viscous damping coefficient. Finally, the quality factor (*Q*) is determined by Equation (11), in which ω denotes angular frequency, and *M* is the moving mass.

#### 2.3.5. Genetic Algorithm Optimization

**Figure 3:**
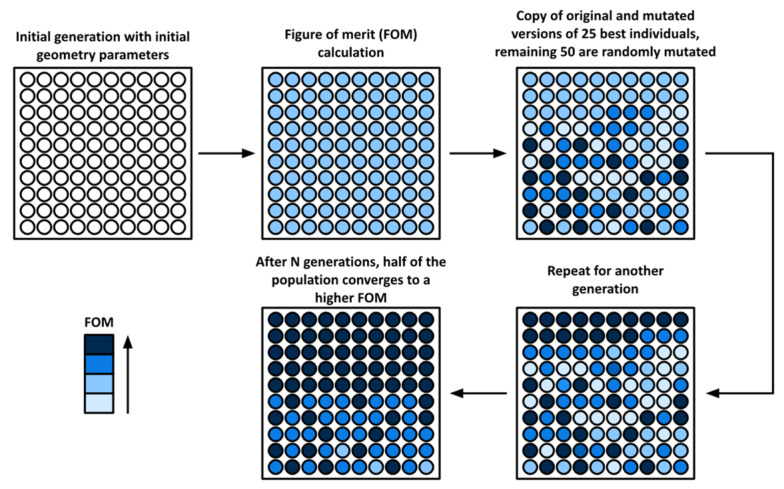
Workflow of the programmed genetic algorithm.

The genetic algorithm (GA) block conducts the electro-mechanical co-optimization. In this study, the genetic algorithm was developed with the following workflow, as illustrated in Figure 3:

1. At first, GA initializes 100 individuals as the first generation with the initial geometric parameters listed;
2. A calculation of each individual Figure of Merit (FOM) is then executed—in the first generation, this attribute is equal for all individuals;
3. For the individuals of the next generation, both an integral copy and mutated copy of the 25 best individuals in the first generation are included and the remaining 50 devices are randomly generated;
4. Process 3 is repeated for a number of generations—until half of the population converges to a high-performance FOM and an individual is selected.

## 3. Results

### 3.1. Case Study 1: MEMS Capacitive Accelerometer

**Figure 4:**
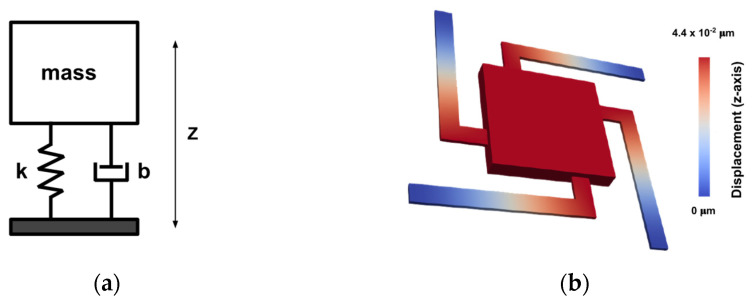
(**a**) Mass-spring-damper model of the MEMS accelerometer; (**b**) the MEMS accelerometer mode shape corresponding to the natural frequency of 3284 Hz.

To show the effectiveness of the developed software, as the first demonstration, an open-loop capacitive MEMS accelerometer is designed, simulated, and optimized. This device comprises a proof-mass suspended by four beams above the substrate. The accelerometer is designed to measure an acceleration in the *z*-axis by detecting the displacement of the proof-mass in the *z*-axis, with a mass-spring-damper model illustrated in Figure 4a.

To detect the capacitance change introduced by the acceleration input, the proof-mass is located between two electrodes with an overlap area equal to the proof-mass surface area. The initial distance between the proof-mass and the electrodes changes when the proof mass experiences a displacement caused by an acceleration.

The MEMS accelerometer structure and its fundamental resonant frequency is illustrated in Figure 4b, with geometric parameters listed in Table 1. The device comprises four *L-shaped* beams connected to the proof-mass on one end and fixed on the other, which suspend the proof-mass above the substrate.

#### 3.1.1. Optimization Results

**Figure 5:**
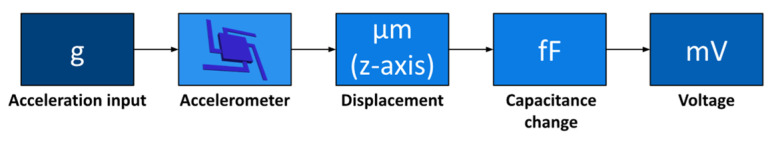
The system-level model of the MEMS accelerometer.

The simulation of the MEMS capacitive accelerometer is based on the flow chart illustrated in Figure 5.

The MEMS accelerometer generates a detectable capacitance change, which is then read by a capacitance-to-voltage circuit, governed by Equation (6). For the simulated accelerometer, *V_DD_* is defined as 5 V, *V_CM_* = 2.5 V, and *C_int_* = 300 fF.

**Figure 6:**
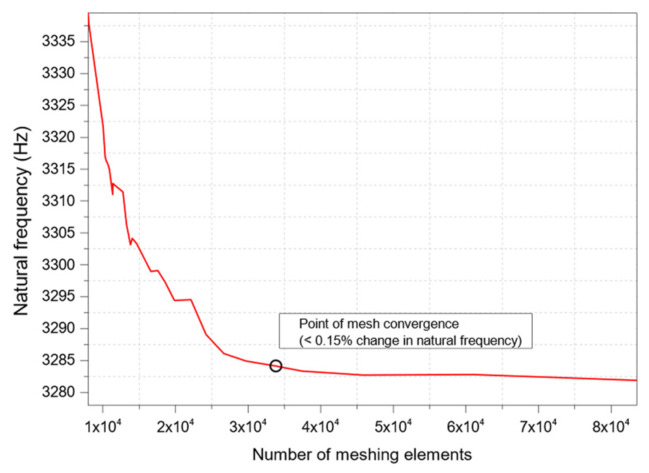
Mesh convergence study for MEMS accelerometer.

The solution of a PDE is strongly related to the density of the mesh. It is, therefore, necessary to perform a mesh convergence study. In this case, the natural frequency is analyzed with different numbers of meshing elements in the suspension beams. As observed in Figure 6, the meshing elements reached the optimal number at 33,824—after this, for the next 6 data points, the variation in the first frequency mode becomes less than 0.15%. Thus, the remaining simulation and optimization process use a number of elements of 33,824 as the optimized meshing element size.

The genetic algorithm optimization process described in Section 2.3.5 is applied to this device for 6 generations, with a FOM defined by Equation (12).

(12) $$\mathrm{FOM}=0.5\cdot Sensitivity\;\left(\frac{\mathrm{mV}}{g}\right)+0.15\cdot \frac{Frequency\;\left(\mathrm{Hz}\right)}{10}+0.35\cdot Q_{factor}\cdot 100$$

In this equation, *Sensitivity* stands for the output voltage when an acceleration of 1 g is applied, *Frequency* is the resonant frequency and *Q_factor_* denotes the quality factor of the sensing mechanism under the atmosphere air pressure. The FOM is comprised by weighted elements—allowing the designers to choose the weight of each performance parameter according to the projected application of the MEMS device.

**Figure 7:**
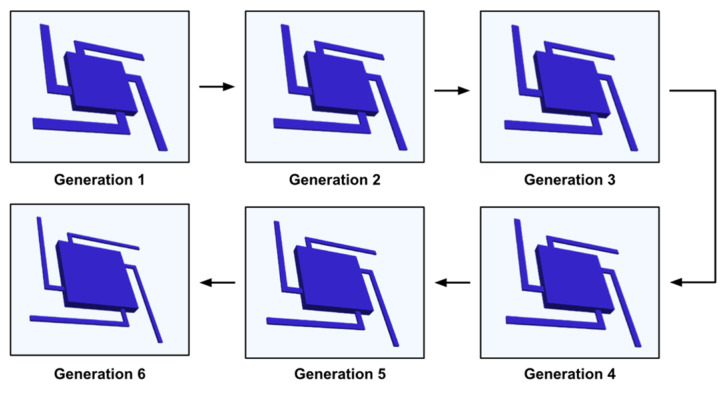
Evolution of the MEMS accelerometer through the 6 generations optimization of the GA. After 6 generations, the suspension beam width is reduced and the proof mass is enlarged.

Within 6 generations (100 individuals in each generation), the GA altered the chosen initial geometric parameters: proof-mass length and suspension beam width and obtained an optimized device. The geometric changes and performance are listed in Table 2. The evolution of the device in Figure 7 illustrates the algorithm tends to reduce the suspension beam width and enlarge the proof-mass size. That increases the sensitivity of the MEMS accelerometer by 193.77% (due to lower stiffness in the suspension system combined with a larger proof-mass) but decreases the resonant frequency by 42.54% and the quality factor by 51.51%. Finally, the FOM is increased by 26.79% after the optimization process.

To verify the accuracy of the FEM analysis performed by the proposed software, a COMSOL simulation of the same device was made. The natural frequency of the MEMS accelerometer obtained by COMSOL was 1897.15 Hz, while the natural frequency of the MEMS accelerometer obtained by the proposed software was 1887.9 Hz. The relative difference of the two simulated natural frequencies was 0.5%, proving the accuracy of the FEM analysis performed by the proposed software.

### 3.2. Case Study 2: Linear MEMS Vibratory Gyroscope

The second demonstrator of the proposed software was a MEMS vibratory gyroscope, reproduced from [22]. This device featured a drive frame implemented to nest the proof-mass and thus decouples the drive and sense motion. A *u-beam* suspension system was put together to ensure that both the drive and sense motion only deflect in the correct direction.

The MEMS gyroscope maintains an oscillation in the drive axis and experienced a Coriolis force under an angular-rate input. The Coriolis force will result in an energy transfer from the drive axis to the sense axis which occurs in the form of a proof-mass movement along this axis.

**Figure 8:**
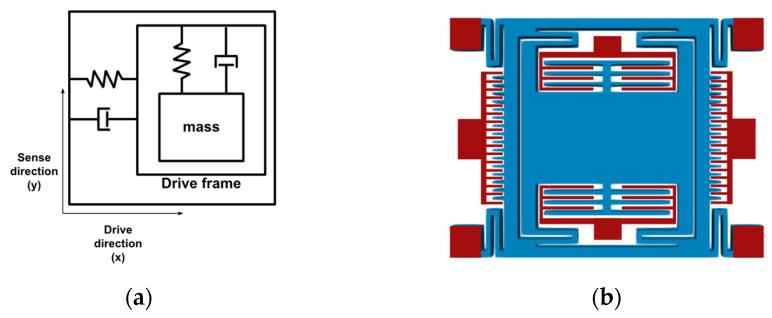
(**a**) Mass-spring-damper model of MEMS gyroscope design; (**b**) linear vibratory MEMS gyroscope design [22].

The geometry built by the software is shown in Figure 8b, with initial geometric parameters listed in Table 3. This design comprises eight anchors, illustrated in red: suspension *u-beams* anchors, stationary electrodes for sensing, and stationary drive electrodes.

The *u-beams* are designed to perform as a suspension system that keeps the proof-mass above the substrate. The four drive frame beams allow movement of the device along the *x*-axis (the drive direction), and the four beams that connect the proof-mass to the drive frame facilitate a displacement by the *y*-axis (the sense direction).

In this gyroscope, drive oscillation along the *x*-axis was actuated through the lateral electrodes shown in Figure 8b. Differential capacitive sensing was used in the device.

#### Optimization Results

**Figure 9:**
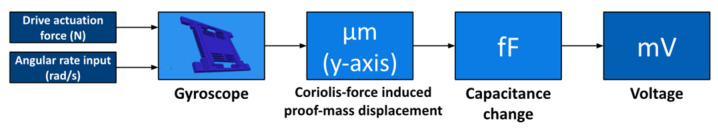
Linear vibratory MEMS gyroscope system-level model.

The simulation and optimization of the vibratory MEMS gyroscope is based on the system-level model illustrated in Figure 9.

The software calculates the drive amplitude of the MEMS device with the drive actuation force governed by Equation (5) and damping (in this mechanism, slide-film damping is the most prominent damping factor). For this actuation mechanism, a DC voltage of 8 V and an AC voltage of 4 V are applied.

Due to driving velocity along the *x*-axis and the angular rate input, the gyroscope experiences a Coriolis force along the *y*-axis, resulting in a displacement of the proof-mass in *y*-axis. This displacement in *y*-axis causes a detectable capacitance change, described by Equations (4) and (6). For the simulated gyroscope, *V_DD_* is defined as 5 V, *V_CM_ =* 2.5 V and *C_int_* = 100 fF.

**Figure 10:**
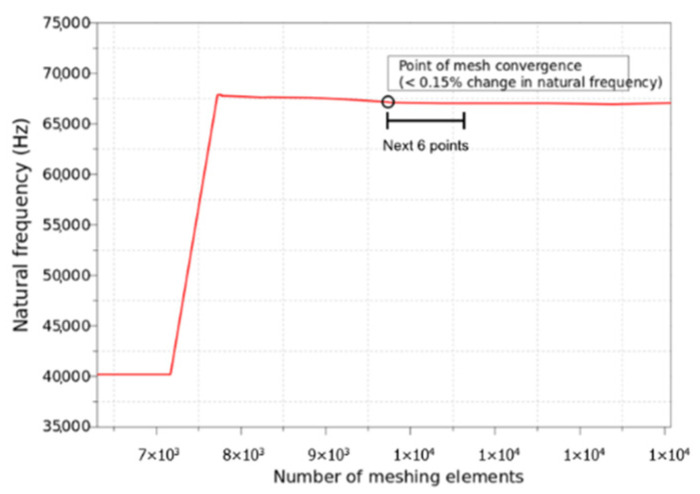
Mesh convergence study for MEMS gyroscope simulation.

For this gyroscope, the first frequency mode is analyzed with different numbers of meshing elements. As observed in Figure 10, the meshing elements reached the optimal number of 9737—after this, for the next 6 data points, the variation in the first frequency mode is less than 0.15%. Thus, the remaining simulation and optimization process will use a number of elements of 9737 as the optimized meshing element size.

The GA optimization process is applied to the MEMS gyroscope for 6 generations. The FOM is defined by Equation (13).

(13) $$\mathrm{FOM}=\left(Sensitivity\;\left(\frac{\mathrm{mV}}{\mathrm{rad}\cdot s^{-1}}\right)\cdot \frac{1}{\Delta f}\cdot Q_{sense}\right)\cdot 10^{6}$$

In this equation, *Sensitivity* is the output voltage when the device is subjected to an angular rate of 1 rad·s^−1^ in the *z*-axis, Δ*f* denotes the difference between drive and sense frequency (influences the mechanical gain in the sense mode to the input angular rate, stated in Equation (14)). Lastly, *Q_sense_* represents the sense mode quality factor. This FOM was presented in a multiplication fashion without weighted elements, this is useful when the designer is not sure which performance parameters are the most important ones.

(14) $$y_{0}=\Omega_{z}\frac{M_{C}\cdot \omega_{D}}{M_{S}\cdot \omega_{S}^{2}}\cdot \frac{2\cdot x_{0}}{\sqrt{\left[1-{\left(\frac{\omega_{D}}{\omega_{S}}\right)}^{2}\right]+{\left[\frac{1}{Q_{sense}}\left(\frac{\omega_{D}}{\omega_{S}}\right)\right]}^{2}}}$$

**Figure 11:**
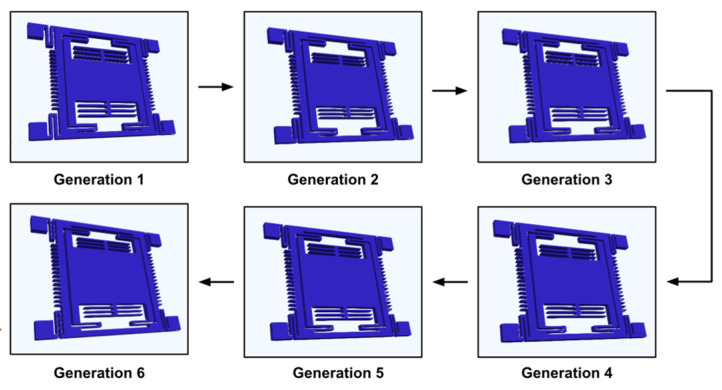
Evolution of the MEMS gyroscope through the six generations of the GA. During the optimization, the proof-mass became larger while the *u-beams*, the sense comb fingers, and the proof-mass frame became thinner.

During the six generations, the GA changed the geometric parameters to find the optimal device. The geometric parameters and performance are listed in Table 4. The geometric parameters were the suspension beam’s width and length, and sense comb fingers’ width. The evolution of the optimization is illustrated in Figure 11.

**Figure 12:**
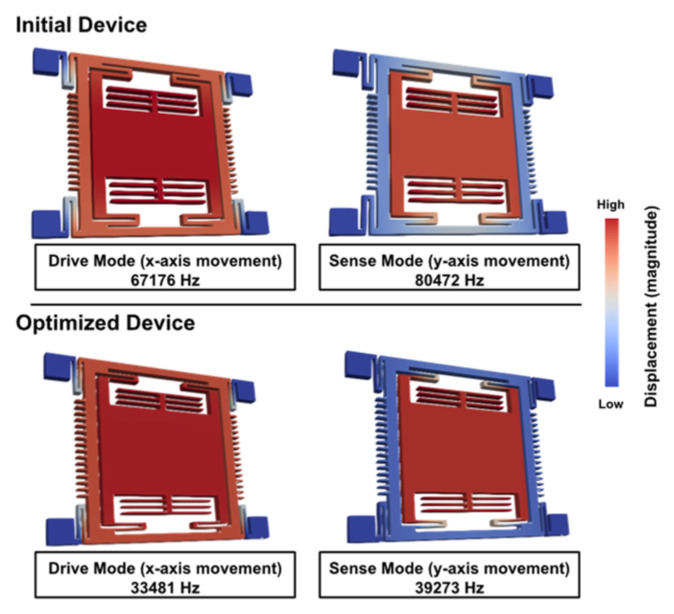
Mode shapes of initial and optimized MEMS gyroscope.

The suspension beam width of the optimal design is much smaller than that of the initial design and the proof-mass size of the optimal design is much larger than that of the initial design. These two geometric changes lead to an increase in sensitivity. Moreover, after 6 generations, the drive and sense mode frequencies as well as the frequency split were significantly reduced. A comparison between frequency modes of the initial and optimized design is shown in Figure 12.

The mode shapes of the MEMS gyro are illustrated in Figure 12. In the optimized design, the undesired *y*-axis movement in the drive frame is reduced, while the desired proof-mass displacement is slightly enhanced. The performance of the initial and optimized design is listed in Table 4. The sensitivity increased by 420.9% and the frequency mismatch decreased by 56.44%. The FOM was improved by 83.17%. However, the quality factor of the sense mode was decreased by 50.31%, this is explained by the decrease in resonant frequency which is inversely correlated with the quality factor.

## 4. Discussion

The software can build geometries of relative complexity, with *Pygmsh* Python library. Although the most complex geometric operations, such as filet, chamfer, Bezier curve, and arrays are not available, most MEMS inertial sensors can be built by the software through coding. When compared with commercial multiphysics tools, the designed software falls short in its ability to create freeform designs and the lack of graphical interface, which makes it less user-friendly.

The geometry building block passes its parameters and meshes to the FEM part of the program, which can conduct modal analysis and displacement. When compared with COMSOL, only a 0.5% difference in the first frequency mode is reported in Section 3.1.1. COMSOL is a multiphysics software, and so it comprises hundreds of different FEM simulations for different physical domains and phenomena. The software described here, in its current state, can only evaluate displacement and eigenfrequency, but users can easily study different physical domains by inserting the correct equations into the script, referring to the FEnICS documentation [13]. The damping script takes into account the geometric parameters, calculating the squeeze film and slide film damping coefficient, and the quality factor. Finally, the implemented genetic algorithm takes the performance of the devices and proceeds to achieve an electro-mechanical co-optimized design in the end. To demonstrate the effectiveness of the proposed software, two MEMS inertial sensors were designed, simulated, and optimized.

Besides, this software offers the possibility of parallel processing, allowing the computer to process long processes faster and more efficiently.

## 5. Conclusions

This study presented a novel electro-mechanical co-optimization methodology for MEMS inertial sensors, entirely based on open source Python. A software comprising geometry design, a FEM simulation, damping calculation, electronic domain calculation, and a GA optimization process, was developed and applied to two MEMS inertial sensors as demonstrators. The developed Python program is a powerful tool that provides designers with limitless customization freedom, presents engineers with complete control of all steps in simulation and optimization and allows efficient management of computational resources according to specific research goals.

The next steps for this software will be the implementation of transient and closed-loop system simulation and optimization. In addition, thermoelastic damping will be added to the damping study block of the system as well as a non-linearity calculation of MEMS devices.

## Figures and Tables

**Table 1:** Initial geometric parameters of the MEMS accelerometer.

Parameter	Value
Suspension beam width	350 µm
Suspension beam length	3300 µm
Beam thickness	69 µm
Small beam length	500 µm
Proof-mass length	2400 µm
Proof-mass thickness	320 µm
Distance proof-mass/electrodes	22 µm

**Table 2:** Geometric and performance of initial and final accelerometer.

Parameter	Initial	Final	Relative Change
Suspension beam width (µm)	350	152	−56.57%
Proof-mass length (µm)	2400	2613	8.15%
Sensitivity (mV/g)	80.747	237.210	193.77%
Frequency (Hz)	3284	1887	−42.54%
*Q_factor_*	0.794	0.385	−51.51%
FOM	117.42	160.38	26.79%

**Table 3:** Initial geometric parameters of MEMS gyroscope.

Parameter	Value
Suspension beam width	20 µm
Suspension beam length	194 µm
Drive frame length	970 µm
Proof-mass lateral beam width	60 µm
Proof-mass lateral beam length	430 µm
Comb finger width	14 µm
Drive comb finger length	48 µm
Sense comb finger length	243 µm
Thickness	50 µm

**Table 4:** Geometric and performance parameters of initial and optimized gyroscope.

Parameter	Initial	Final	Relative Change
Suspension beam width (µm)	20	9	−55.00%
Proof-mass frame width (µm)	430	395	−8.14%
Proof-mass frame length (µm)	60	54	−10.00%
Proof-mass width (µm)	290	309	6.15%
Proof-mass length (µm)	220	240	8.33%
Sense finger width (µm)	14	9	−35.71%
*Sensitivity* (mV/rad∙s^−1^)	1.546	8.054	420.9%
Δ*f*	13,296	5792	−56.44%
*Q_sense_*	17.47	8.68	−50.31%
FOM	2031.3	12,069.9	83.17%

## Data Availability

Not applicable.
